# Effect of AI empathy perception on employees' prosocial behavior: mediating role of warmth and moderating role of AI anthropomorphism

**DOI:** 10.3389/fpsyg.2025.1706756

**Published:** 2026-01-12

**Authors:** Jinli Xue, Yuhan Liu, Zengfang Ren, Yige Wu

**Affiliations:** 1 Center for Energy Economics Research, School of Business Administration, Henan Polytechnic University, Jiaozuo, China; 2 Qingdao Institute of technology, School of Economics and Management, Qingdao, China

**Keywords:** AI, AI anthropomorphism, empathy perception, prosocial behavior, warmth

## Abstract

With the rapid advancement of AI technology, an increasing number of organizations are introducing AI as formal employees in the workplace. By conducting hypothesis testing on 400 sample data from eight highly digitalized companies in China, the findings show that the perception of AI empathy can enhance employees' prosocial behavior, with warmth serving as a mediating factor. Furthermore, AI anthropomorphism moderates the effect of AI empathy perception on employees' prosocial behavior through warmth: the higher the degree of AI anthropomorphism, the greater the positive impact of AI empathy perception on warmth, which in turn positively moderates the effect of AI empathy perception on human employees' prosocial behavior. This paper constructs a pathway of “technological environment-emotional cognition-behavioral activation,” expanding the research perspective on behavior and emotional cognition within the domain of human-computer interaction. It elevates AI's emotional interaction to the level of organizational behavior management, promoting a shift from human-machine collaboration to human-to-human collaboration.

## 1 Introduction

Empathy, a complex interplay of human emotion and cognition, has become a focal point in artificial intelligence (AI) research as technological advancements enable AI to move beyond passive tools toward active social collaborators (Dennis et al., 2023). Unlike traditional AI, Social Emotional Artificial Intelligence (SEAI) possesses emotional recognition and response capabilities, allowing it to engage in meaningful emotional interactions with humans—filling roles that demand emotional sensitivity (Fiske et al., 2007; Peng et al., 2022). This shift from “task aid” to “emotional partner” has garnered attention from business leaders, as AI's integration into teams represents a transformative change in workplace dynamics (Menon and Dubé, 2007; Trainer et al., 2020).

Notably, AI empathy perception is conceptually distinct from AI anthropomorphism: while anthropomorphism refers to the general attribution of human-like qualities to machines, AI empathy perception specifically denotes the perceived capacity of AI to exhibit emotional attunement, moral concern, and communicative responsiveness toward humans. This distinction addresses a critical gap in prior research, which has primarily focused on anthropomorphism or trust in human-AI interactions (e.g., Caić et al., 2020; Kim et al., 2019) but has not explicitly defined or examined AI empathy perception as a unique construct.

Existing studies on AI and prosocial behavior offer conflicting insights: Cheng et al. (2024) and Granulo et al. (2024) found that algorithmic management reduces prosocial motivations by depersonalizing colleagues, but this effect is mitigated by workplace social relationships. However, these studies frame AI as a managerial tool, not an equal emotional collaborator. Our research fills this void by focusing on SEAI as a non-managerial, emotionally expressive partner—eliminating the depersonalizing context of algorithmic management—and investigating how employees' perception of AI's empathy (rather than mere anthropomorphism) influences their prosocial behavior.

Guided by social cognition theory, we propose a theoretical framework that links AI empathy perception to employee prosocial behavior through the mediation of perceived warmth, with AI anthropomorphism moderating this process. Warmth, a core dimension of social cognition (Fiske, 2018), refers to employees' emotional cognitive response to AI's empathy—encompassing perceptions of friendliness, sincerity, and helpfulness (Zhao et al., 2024). While prior research has linked AI warmth to attitudinal outcomes (Caić et al., 2020; Song et al., 2022), the mechanism by which AI empathy perception (distinct from anthropomorphism) triggers warmth and subsequent prosocial behavior remains unexamined.

Our study addresses three key research objectives: (1) Empirically test the direct effect of AI empathy perception on employee prosocial behavior, challenging the traditional view of AI as solely a “task collaborator” and highlighting its role as an “emotional interactor”; (2) Explore the mediating role of perceived warmth in the relationship between AI empathy perception and prosocial behavior, clarifying that warmth here reflects employees' emotional cognitive responses to AI (not just AI's inherent traits); (3) Examine the moderating role of AI anthropomorphism, investigating how human-like features of AI amplify or weaken the mediated path from empathy perception to prosocial behavior via warmth.

By distinguishing AI empathy perception from anthropomorphism and unpacking the mediating and moderating mechanisms, this research advances understanding of human-AI emotional interactions in the workplace—addressing theoretical gaps in social cognition and AI management research while providing practical implications for organizations integrating SEAI into teams.

## 2 Literature review and theoretical framework

### 2.1 AI empathy perception

Modern enterprise management emphasizes a human-centered approach, where empathy between individuals is a critical dimension for coordinating interpersonal relationships within organizations. It has become an important indicator for predicting employees' prosocial behaviors (Zaki, 2020). Therefore, empathy plays a crucial role in understanding employee interactions in organizational management (Liu et al., 2023). The care and attention shown between organizational employees help them feel that their peers are warm and personalized, which triggers empathetic responses (Preston and De Waal, 2002). The understanding and feedback from empathizers typically increase the trust and confidence of observers, thereby altering their behavior (Parasuraman et al., 1988). Recent advancements in artificial intelligence have made it possible for AI to interact fully with organizational members, opening up new avenues for studying employee behavior. Emotion Recognition Systems (ERS), an emerging technology in AI, allows machines to recognize human emotions by learning various data patterns (Mohd Yamin et al., 2023), using affective computing to understand and respond to humans (Landowska, 2019). Yang et al. (2023) discussed in their research on artificial intelligence and management transformation that, relying on the cloud-edge network architecture of the internet, AI can continually learn about each user's knowledge, personality, habits, and thinking during communication usage. Based on this, emotion recognition is performed on interactants through affective computing, analyzing elements such as voice, text, and images. Subsequently, empathic responses are provided through behavioral empathy. This means that when you feel frustrated, your AI may offer more encouragement and support, and when you are excited, it may engage more enthusiastically with you. Affective computing, introduced by Picard (2003), aims to give machines emotional characteristics and emotional interaction capabilities, enabling them to engage in natural and dynamic interactions with humans. For AI to perform affective computing, it requires scene perception and the recognition and analysis of facial expressions, body language, or tone of voice to identify emotions consistent with the interactant. By computing the correlation between emotion types and the context of the situation, AI can understand the emotion and select appropriate behavior for the current context, thus generating an “empathic response” to humans (Lim and Okuno, 2015).

The emergence of AI empathic responses may be one of the key factors triggering further organizational transformation in the business environment. For instance, AI is no longer merely an auxiliary intelligent tool; instead, it interacts with humans as a fully autonomous primary collaborator, competitor, or assistant within various organizational contexts (Harris-Watson et al., 2023). However, the relationship between humans and AI is not only contingent upon whether AI will truly possess empathic abilities, but more importantly, on how humans perceive AI's capacity for empathy (Pataranutaporn et al., 2023). Previous research has shown that, in addition to the efficiency and appeal brought about by AI, social groups also play a crucial role in the acceptance of AI by interactants (Gursoy et al., 2019; Kim and Baek, 2018; Pelau et al., 2021). According to the Computers as Social Actors theory, viewing AI devices as social actors prompts interactants to engage with them in ways similar to human interaction (Heerink et al., 2010; Nass et al., 1995). By extension, we infer that employees' perceptions of AI empathy during interactions may parallel their perceptions of empathy in human interactions.

### 2.2 Warmth

Warmth is defined as employees' emotional and cognitive response to the empathic expressions of AI—specifically contextualized within AI-employee interaction scenarios—rooted in Fiske et al.'s (2007) warmth–competence model. It encompasses subjective perceptions of friendliness, sincerity, and helpfulness formed through employees' evaluation of AI's positive intentions (Cuddy et al., 2011; Jiang et al., 2023). Importantly, this conceptualization distinguishes warmth from AI's inherent technical attributes: it is not an intrinsic trait of AI systems but a psychological reaction triggered when employees perceive AI's contextually adaptive empathic behaviors. For large language models (LLMs)—a representative type of AI widely adopted in workplace interactions—concrete expressions of warmth include: (1) validating employees' emotional states related to work (e.g., “I understand you're feeling pressured by the tight deadline; let's break down the task step by step”); (2) providing responsive and patient communication when addressing complex or ambiguous work-related queries (e.g., elaborating on technical concepts with practical examples rather than using jargon); and (3) proactively offering tailored support aligned with employees' implicit needs (e.g., suggesting time-management tools after an employee mentions struggling with multitasking, or sharing industry best practices relevant to their ongoing projects).

Researchers have emphasized that perceived warmth strongly predicts future interpersonal relationship outcomes (Eisenbruch and Krasnow, 2022), as it fosters a sense of social connection that drives behavioral change. Specifically, employees who perceive care and understanding from AI are motivated to reciprocate through prosocial actions, given that warmth activates neural and bodily representations associated with altruism (Gómez-León, 2022). Furthermore, Newton et al. (2022) found that when selecting collaborative partners, team members tend to prioritize individuals (or AI systems, by extension) who exhibit warmth by building relational bridges in the work environment. Warmth has consistently played a dominant role in shaping willingness to collaborate, with people universally prioritizing warmth over ability across contexts (Cuddy et al., 2011)—a pattern that holds particular relevance in AI–employee interactions. This is because perceived ability primarily reflects judgments of whether an individual (or AI) can effectively implement their intentions, often measured by traits such as intelligence, efficiency, and technical skill. Notably, AI's work capabilities—bolstered by technological advancements—have been widely recognized across various professional fields. In contrast, warmth perception is rooted in the evaluation of positive intentions, and warmth-related behaviors inherently carry altruistic attributes (Jiang et al., 2023), making it a unique and critical construct in understanding how employees engage with AI in the workplace.

### 2.3 Prosocial behavior

Prosocial behavior refers to social actions consciously performed by individuals in interpersonal interaction contexts within real and effective natural environments, aimed at benefiting others or maintaining collective interests (Carlo, 2013; Boxer et al., 2004). Zhang et al. (2024) classified the concept of prosocial behavior into two representations: the classical theoretical representation and the prototype theoretical representation. Their research suggests that the classical theoretical representation of prosocial behavior tends to emphasize the purely altruistic nature of such behaviors, focusing on the impact of the behavioral outcome on the recipient. In contrast, the prototype theoretical representation of prosocial behavior, based on the actor's own cognition, views prosocial behavior as a social action that arises when individuals maintain friendly and positive relationships with others and promote collective interests. This form of prosocial behavior is seen as a means through which both parties can benefit, and it is characterized by reciprocity and social appropriateness. For example, scholars have described that, firstly, for individuals, prosocial behavior functions to enhance self-life satisfaction and a sense of meaning in life, helping individuals to positively cope with negative emotions and maintain mental health (Tian et al., 2023; Miles et al., 2021; Nelson et al., 2016). Secondly, in the context of interpersonal relationships, prosocial behavior can help improve social interactions, enhance relationships, and facilitate interpersonal adaptation and harmony (Wang et al., 2019). Lastly, from a societal perspective, prosocial behavior is both influenced by social factors and related to social responsibility and public welfare, serving as the foundation for the construction and development of a harmonious society (Miller et al., 2015). Our study suggests that when employees perceive warmth generated by AI empathy, it fosters employee development and growth. Additionally, employees are likely to engage in altruistic and reciprocal prosocial behaviors—such as benefiting others, enhancing their own satisfaction and well-being, and promoting teamwork and collaboration—through the transmission of this warmth.

### 2.4 AI Anthropomorphism

Based on the anthropomorphization definition, AI anthropomorphization refers to the process of attributing a series of human-specific characteristics to AI, serving as a method or approach of design or control (Yao et al., 2022). Currently, due to advancements in artificial intelligence algorithms and deep learning technologies, much scientific interest focuses on transferring human traits, such as empathy, to computer systems. Socially emotional AI applies certain human social-emotional traits to artificial intelligence. For example, social-emotional robots may vary in form or function, but they share common characteristics in certain aspects (Woo et al., 2021). These types of AI can detect the presence of humans, engage in social interactions with them, express their own “emotional state,” and respond to the emotional state of the conversational partner. Additionally, they are capable of communicating in a natural, human-like manner, including non-verbal communication, such as gestures, posture, facial expressions, and any other intuitive form of interaction (Gómez-León, 2022). This can also be referred to as AI anthropomorphization. AI anthropomorphization can be manifested in the form of physical appearance or behavior and is widely applied in fields like psychology, marketing, and computer science (Schanke et al., 2021). Firstly, AI physical anthropomorphization refers to the similarity in form between algorithm-enabled robots and humans (Miao et al., 2022). Scholars have pointed out that physical anthropomorphization can influence people's acceptance of AI recommendations (Mende et al., 2019). For example, when a robot's appearance resembles that of a human, people tend to perceive it as having the ability to understand emotions similarly to humans and are more likely to accept its suggestions (Longoni and Cian, 2022; Yam et al., 2021). Additionally, people may assume that robots share the same cognitive patterns as humans, which can enhance their attitude toward and willingness to use robot services (Prahl and Van Swol, 2017). Secondly, behavioral anthropomorphization refers to the similarity in behavior between algorithm-enabled robots and humans (Glikson and Woolley, 2020), which directly affects people's perception of the internal logic of AI algorithms. Research on the impact of AI anthropomorphization on service interaction and outcomes has shown that robots with physical and behavioral anthropomorphization create a sense of automation and social presence, which, through social perceptions (warmth, capability), mediates and enhances customer satisfaction and engagement (Van Doorn et al., 2017). In hotel service robots, AI machines not only replace human service functions (such as greeting, service guidance, food delivery, and other simple tasks), but also engage in human-like interactions with customers (such as playful interactions, reducing service wait anxiety, and responding to customers' facial expressions and language). Additionally, AI can reduce stress and loneliness by providing comfort, companionship, and emotional support, thereby enhancing the enjoyment of the service experience and the stability of service quality, ultimately improving customer well-being (Robinson et al., 2013). As mentioned above, we believe that AI with physical and behavioral anthropomorphization can better bridge the gap between AI and humans during interactions, with human-like appearance, verbal expressions, and the smoothness of behavior all contributing to enhancing humans' social perception.

### 2.5 Theoretical model and hypotheses

Guided by social cognition theory, we propose a moderated mediation model where: (1) AI empathy perception directly promotes employees' prosocial behavior; (2) warmth (employees' emotional response to AI empathy) mediates this relationship; (3) AI anthropomorphism moderates the direct effect of AI empathy perception on warmth; and (4) AI anthropomorphism moderates the indirect effect of AI empathy perception on prosocial behavior via warmth. Hypotheses:

- H1: AI empathy perception positively promotes an increase in employees' prosocial behaviors.
- H2: Warmth mediates the positive relationship between AI empathy perception and employees' prosocial behavior.
- H3: AI anthropomorphism magnifies the positive relationship between AI empathy perception and warmth, such that the relationship is stronger when AI anthropomorphism is high rather than low.
- H4: AI anthropomorphism positively moderates the relationship between AI empathy perception and employee prosocial behavior through warmth, meaning that an increase in AI anthropomorphism can enhance the warmth generated by AI empathy perception, thereby increasing prosocial behavior.

**Figure 1:**
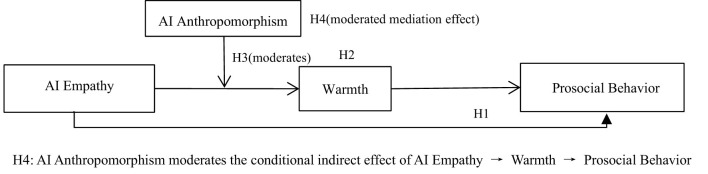
Proposed model.

The proposed model is shown in Figure 1.

## 3 Methods

### 3.1 Participants and procedure

This research utilized an online survey to gather sample data. Initially, we reached out to HR managers from various companies and secured the approval and backing of their company leaders. Second, we explained the purpose of the survey to employees in their respective WeChat groups, requesting their consent and cooperation for the distribution and collection of the online questionnaires. The data collection took place on February 28, 2024, March 29, 2024, and April 29, 2024 (three times). The respondents consisted of salespeople, researchers, administrative staff, production, and operations personnel from eight companies in China. Since the integration of AI members with empathy is a relatively new and transformative organizational management strategy, requiring companies to adopt flexible management approaches and a high level of digitalization, the chosen companies were primarily those with advanced digital technologies and AI utilization. All respondents had some level of collaboration with AI in their work processes.

To ensure data validity, this study adopted a time-lagged measurement approach, collecting data at three separate time points: Time 1 (T1), Time 2 (T2), and Time 3 (T3). At T1, data was gathered on human employees' perceptions of AI empathy within organizations that had implemented empathetic AI members, along with demographic variables such as gender, age, tenure, education level, job type, and organizational characteristics. A total of 503 questionnaires were distributed to employees across the aforementioned eight enterprises. After excluding invalid responses (e.g., duplicate questionnaires from the same device ID, failure to pass screening questions, or extreme/abnormal responses), 465 valid questionnaires were collected, yielding an effective response rate of 92.45%. Additionally, at T1, employee ID numbers and the last digits of their phone numbers were recorded to accurately match participants in subsequent data collection phases. One month later, at T2, measurements were taken for warmth and AI anthropomorphism. Questionnaires were distributed to the 465 participants who had completed valid questionnaires at T1, and 431 valid responses were collected, yielding an effective response rate of 92.69%. Another month later, at T3, measurements were taken for employees' prosocial behavior. Questionnaires were distributed to the 431 participants who had completed valid questionnaires at T2, and 407 valid responses were collected, yielding an effective response rate of 94.43%. From the total sample of 407, 7 invalid samples were excluded, resulting in 400 valid samples (60.8% female, 39.3% male; mean age = 31.17, SD = 7.67; mean tenure = 7.72, SD = 6.67), with an effective response rate of 79.52%. Of these, the majority had a bachelor's degree (66.5%), followed by master's degrees (17.8%). In this study, the proportional distribution of the four functional categories was as follows: Research and Development accounted for 35.0%, Sales personnel for 16.8%, Administrative staff for 25.0%, and Production and Operations for 23.3%. Given that the samples for this study were drawn from companies with advanced digital technologies, and the data were gathered from employees with significant interaction with AI, their understanding and acceptance of the research were generally high.

### 3.2 Measures

The scales utilized in this study were derived from established international literature and were confirmed to be applicable to the local context through our pretest. Professors from the Foreign Language Department and foreign experts were involved in this process. Initially, the English scales were translated using the back-translation method. Then, the Chinese versions were re-translated into English using an interactive translation approach. This back-and-forth translation process was repeated several times to ensure both semantic accuracy and fluency, while also maintaining academic rigor. The scales were then distributed to three companies using empathetic AI members for pretesting, ensuring that the content aligned with local language norms. Except for control variables, the manipulation variables were assessed using a five-point Likert scale, where participants were asked to rate the extent to which they agreed with the descriptions on a scale from 1 (strongly disagree) to 5 (strongly agree), based on their personal experiences. Before the survey, we reassured employees that their responses would be anonymous and used solely for academic purposes. We also emphasized the confidentiality of all information to encourage honest and open responses.

#### 3.2.1 AI empathy perception

The five-item scale developed by Kellett et al. (2006) was used and appropriately adapted to the specific context. One of the items is: “I believe AI empathy can share others' feelings of happiness” The Cronbach's alpha for this scale is 0.862.

#### 3.2.2 Warmth

A four-item scale used by Fiske et al. (2002) was employed and appropriately adapted to the specific context. One of the items is: “An AI capable of empathy would be good-natured” The Cronbach's alpha for this scale is 0.836.

#### 3.2.3 AI anthropomorphism

The five-item anthropomorphism scale adapted by Bartneck et al. (2009) was used and appropriately tailored to the specific context. One of the items is: “I believe AI agents should have smooth and graceful movements” The Cronbach's alpha for this scale is 0.842.

#### 3.2.4 Prosocial behavior

A four-item scale developed by Ryan and Connell (1989) and cited by Grant (2008) was used to measure employees' prosocial behavior, with appropriate adaptations to the specific context. One of the items is: “Because I want to help others through my work” The Cronbach's alpha for this scale is 0.810.

Control Variables. To eliminate the potential influence of irrelevant factors on the relationships between the measured variables in this study, we controlled for demographic variables such as gender, age, tenure, education level, job type, and organizational characteristics.

## 4 Results

### 4.1 Analysis of common method bias

In this study, factor analysis was conducted using SPSS 26.0 to examine whether a single factor could explain most of the variance. The KMO and Bartlett's test results in Table 1 show that the KMO value for sampling adequacy is 0.921, which is greater than 0.9, indicating excellent sample adequacy. The significance level is 0.000 (less than 0.05), suggesting that the correlations in the sample data are significant and suitable for factor analysis. In Table 2, the results show that the eigenvalue of the first factor is 7.257, which accounts for 30.314% of the variance. The eigenvalue of the second factor is 2.005, accounting for 17.140% of the variance. The eigenvalue of the third factor is 1.322, accounting for 10.347% of the variance. The eigenvalue of the fourth factor is 1.082, accounting for 7.012% of the variance. The cumulative variance is 64.813%, indicating that the first four factors explain most of the variance. No single factor dominates the analysis.

**Table 1:** KMO and bartlett's test.

KMO sampling adequacy measure	0.921	
Bartlett's test of sphericity	Approximate chi-square	3417.230
	df	153
	P	0.000

**Table 2:** Harman's single-factor test of total variance explained.

**Component**	**Initial eigenvalues**			**Extracted sum of squared loadings**		
	**Total**	**Variance percentage**	**Cumulative %**	**Total**	**Variance percentage**	**Cumulative %**
1	7.257	30.314	30.314	7.257	30.314	30.314
2	2.005	17.140	47.454	2.005	17.140	47.454
3	1.322	10.347	57.801	1.322	10.347	57.801
4	1.082	7.012	64.813	1.082	7.012	64.813

### 4.2 Confirmatory factor analyses

We conducted confirmatory factor analysis (CFA) using Amos 24.0 to examine the discriminant validity of the variables. Taking the four-factor model as the baseline, three alternative models were constructed: a one-factor model (AI empathy perception + warmth + AI anthropomorphism + prosocial behavior), a two-factor model (AI empathy perception + warmth + AI anthropomorphism, and prosocial behavior), and a three-factor model (AI empathy perception + warmth, AI anthropomorphism, and prosocial behavior). Table 3 presents the CFA results. The data analysis indicated that the four-factor model demonstrated the best fit (χ^2^/df = 1.731, CFI = 0.972, TLI = 0.966, RMSEA = 0.043, SRMR = 0.039) compared to the other models, with all indices meeting acceptable thresholds, suggesting good discriminant validity of the data structure.

**Table 3:** Results of confirmatory factor analyses.

**Model**	**χ^2^**	**df**	**χ^2^/df**	**CFI**	**TLI**	**RMSEA**	**SRMR**
1-Factor model (AIEP+W+AIA+PB)	1043.726	135	7.731	0.727	0.690	0.130	0.096
2-Factor model (AIEP+W+AIA, PB)	661.431	134	4.936	0.841	0.819	0.099	0.068
3-Factor model (AIEP+W, AIA, PB)	424.517	132	3.216	0.912	0.898	0.075	0.054
4-Factor model (AIEP, W, AIA, PB)	223.296	129	1.731	0.972	0.966	0.043	0.039
5-Factor model (4-factor +CMB)	153.823	111	1.386	0.987	0.982	0.031	0.029

The variables in this study were mean-centered before calculating the interaction terms. AIEP, AI Empathy Perception; W, Warmth; AIA, AI Anthropomorphism; PB, Prosocial Behavior; CMB, Common Method Bias.

To assess the potential impact of the model, we referred to the study by Podsakoff et al. (2003) and added a common method factor to the four-factor model structure, creating a five-factor model. The results presented in Table 3 indicate an improvement in model fit (χ^2^/df = 1.386, CFI = 0.987, TLI = 0.982, RMSEA = 0.031, SRMR = 0.029), suggesting the presence of some degree of common method bias. However, the increase in indices such as CFI and RMSEA was minimal, not exceeding 0.02, implying that common method bias does not significantly affect the validity of the research results.

### 4.3 Descriptive statistics

We conducted descriptive statistics and correlation analysis using SPSS 26.0, with the results presented in Table 4. AI empathy perception (x) was significantly positively correlated with employees' prosocial behavior (y) (r = 0.307, *p* < 0.01); AI empathy perception was significantly positively correlated with warmth (m) (r = 0.618, *p* < 0.01); and warmth was significantly positively correlated with employees' prosocial behavior (r = 0.386, *p* < 0.01). Additionally, the correlation coefficients between the manipulation variables were all below 0.7. The statistical results provide preliminary support for Hypothesis 1 and Hypothesis 2.

**Table 4:** Means, standard deviations, and correlation matrix for key measures.

**Variables**	**1**	**2**	**3**	**4**	**5**	**6**	**7**	**8**
1.Gender	-							
2.Age	0.022	-						
3.Education	0.133^**^	−0.093	-					
4.Seniority	−0.016	0.875^**^	−0.141^**^	-				
5.AI empathy perception	−0.075	0.139^*^	0.122^*^	0.167^**^	-			
6.Warmth	−0.052	0.061	0.097	0.082	0.618^**^	-		
7.AI anthropomorphism	−0.124^*^	0.157^**^	0.046	0.167^**^	0.616^**^	0.560^**^	-	
8.Prosocial behavior	−0.010	0.149^*^	0.072	0.146^**^	0.307^**^	0.386^**^	0.405^**^	-
M	1.610	2.070	3.010	2.560	3.553	3.737	3.303	3.999
SD	0.489	1.007	0.675	1.298	0.843	0.813	0.854	0.721

^*^p < 0.05.

**p < 0.01.

### 4.4 Hypothesis testing

We conducted hierarchical regression analysis using SPSS 26.0 as analytical tools to validate the aforementioned hypotheses (Yoo and Brooks, 2005). The results of the hierarchical regression analysis are shown in Table 5.

**Table 5:** Hierarchical regression results.

**Variables**	**Warmth**				**Prosocial behavior**			
	**Model 1**		**Model 2**		**Model 3**		**Model 4**	
	β	**s.e**	β	**s.e**	β	**s.e**	β	**s.e**
Intercept	1.606	0.222	3.263	0.405	2.799	0.237	2.340	0.243
Gender	−0.014	0.067	0.035	0.062	0.005	0.071	0.009	0.069
Age	−0.025	0.066	−0.042	0.061	0.067	0.071	0.074	0.068
Education	0.026	0.049	0.012	0.045	0.052	0.053	0.045	0.051
Seniority	0.006	0.052	0.001	0.048	0.014	0.055	0.012	0.054
AI empathy perception	0.595^***^	0.039	−0.135	0.106	0.243^***^	0.042	0.073	0.051
AI anthropomorphism			−0.485^**^	0.139				
AI empathy perception^*^AI anthropomorphism			0.209^***^	0.036				
Warmth							0.286^***^	0.052
R^2^	0.383		0.482		0.108		0.172	
ΔR^2^	0.375		0.472		0.097		0.159	
F	48.833^***^		52.054^***^		9.547^***^		13.610^***^	

^*^P < 0.05.

^**^P < 0.01.

^***^P < 0.001.

Firstly, linear regression analysis was performed using SPSS. Model 3 in Table 5 indicates a significant positive effect of AI empathy perception on prosocial behavior (β = 0.243, *p* < 0.001). Thus, Hypothesis 1 was supported.

Next, this study employed the method proposed by Baron and Kenny (1986) to test the mediating effect of warmth in Hypothesis 2. Model 1 showed that AI empathy perception was significantly positively correlated with warmth (β = 0.595, *p* < 0.001). Furthermore, in Model 3, the positive effect of AI empathy perception on employees' prosocial behavior was significant (β = 0.243, *p* < 0.001). However, after adding the mediating variable warmth to the model, the effect of AI empathy perception on employees' prosocial behavior became non-significant (β = 0.073, *p* > 0.05), while warmth had a significant positive effect on employees' prosocial behavior (β = 0.286, *p* < 0.001) in Model 4. This change indicated that warmth fully mediated the relationship between AI empathy perception and employees' prosocial behavior. To further confirm the mediating effect of warmth in Hypothesis 2, bootstrap sampling analysis (bootstrap = 5,000, Wang and Preacher, 2015) was conducted using the PROCESS v4.1 macro in SPSS. The results of the mediation analysis are shown in Table 6. The 95% confidence interval for the total effect was (0.160, 0.325) (excluding 0), with a total effect value of 0.243. The 95% confidence interval for the indirect effect was (0.069, 0.342) (excluding 0), with a fully standardized indirect effect value of 0.199. Meanwhile, the 95% confidence interval for the direct effect was (−0.027, 0.173) (including 0), which means that warmth fully mediated the relationship between AI empathy perception and employees' prosocial behavior. Therefore, Hypothesis 2 was supported.

**Table 6:** Analysis of mediating effect of warmth.

**Effect type**	**Effect**	**s.e**	**Bootstrap 95% CI**		**Ratio of total effect**
			**Lower limit**	**Upper limit**	
Total effect	0.243	0.042	0.160	0.325	
Direct effect	0.073	0.051	−0.027	0.173	0.300
Indirect effect	0.170	0.064	0.056	0.306	0.700
CM indirect effect	0.199	0.070	0.069	0.342	

CM, Completely Standardized.

**Figure 2:**
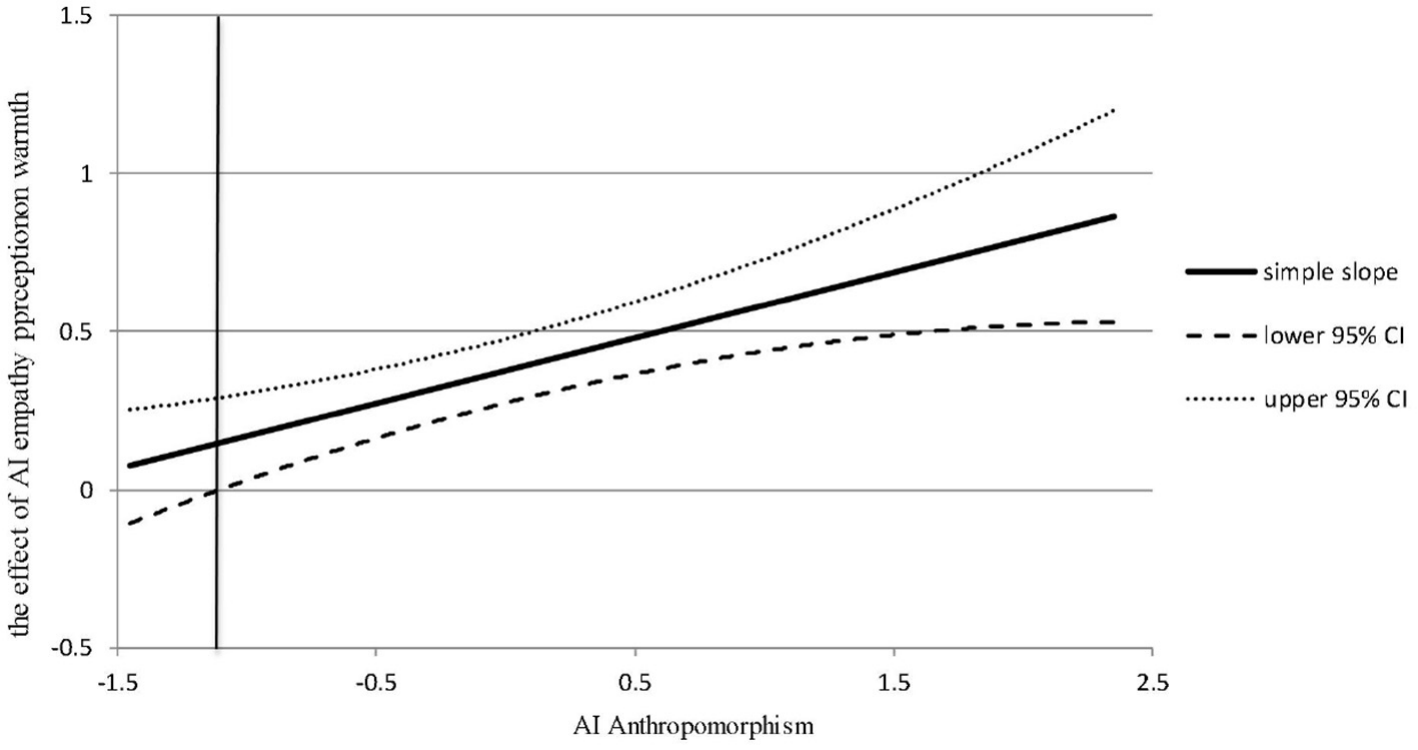
The moderating role of AI anthropomorphism (J-N).

Furthermore, Model 2 in Table 5 showed that the interaction between AI empathy perception and AI anthropomorphism had a significant positive effect on warmth (β = 0.209, *p* < 0.001). This provided preliminary evidence for Hypothesis 3, suggesting that AI anthropomorphism positively moderates the relationship between AI empathy perception and warmth. Subsequently, this study employed the Johnson-Neyman (J-N) method to further explore the moderating effect of AI anthropomorphism, as shown in Figure 2. The J-N method helped uncover additional intrinsic information by providing a simple slope confidence band, thereby addressing the limitations of traditional point-by-point methods in testing moderating effects. As shown in Figure 2, when AI anthropomorphism exceeded −1.172, the confidence interval of the simple slope did not include 0 (95% confidence interval limits). Therefore, the moderating effect of AI anthropomorphism was significant, and the simple slope was greater than 0 and sloped upwards to the right, indicating that when the level of AI anthropomorphism was higher, the positive impact of AI empathy perception on warmth was stronger. Thus, Hypothesis 3 was further supported.

Finally, we employed the bias-corrected non-parametric percentile residual Bootstrap method to further test the moderating effect under two different conditions: high AI anthropomorphism (+1SD) and low AI anthropomorphism (−1SD) (Bjørnson and Dingsøyr, 2008). According to the recommendations of Edwards and Lambert (2007), we used Mplus 8.3 to analyze the moderating effect of AI anthropomorphism (Bootstrap = 5,000). The test results are shown in Table 7. Table 7 revealed that when the level of AI anthropomorphism was high (+1SD), the indirect effect (${\text{P}}_{\text{XM}}^{*}$P_MY_) was β = 0.210, 95% CI (0.092, 0.337), excluding 0, indicating a significant indirect effect. When the level of AI anthropomorphism was low (-1SD), the indirect effect (${\text{P}}_{\text{XM}}^{*}$P_MY_) was β = 0.108, 95% CI (0.038, 0.227), also excluding 0. Furthermore, the difference in the indirect effect across different levels of AI anthropomorphism was significant [β = 0.102, 95% CI (0.032, 0.221)]. This suggests that AI anthropomorphism significantly moderates the indirect effect of AI empathy perception on employees' prosocial behavior through warmth. Therefore, Hypothesis 4 was supported.

**Table 7:** Analysis results of moderated mediating effect.

**Variable**		**First stage**	**Second stage**	**Direct effect**	**Indirect effect**	**Total effect**
		**X**→**M**	**M**→**Y**	**X**→**Y**	(${\text{P}}_{\text{XM}}^{*}$ **P**_MY_ **)**	**P**_XY_+(${\text{P}}_{\text{XM}}^{*}$P_MY_**)**
High anthropomorphism	Estimate	0.733^***^	0.400^***^	0.218^*^	0.210^**^	0.290^***^
(+1s.d.)	95%CI	(0.527, 0.929)	(0.188, 0.610)	(0.053, 0.382)	(0.092, 0.337)	(0.158, 0.432)
Low anthropomorphism	Estimate	0.376^***^	0.218^*^	0.035	0.108^*^	0.188^**^
(−1s.d.)	95%CI	(0.179, 0.558)	(0.021, 0.400)	(−0.165, 0.212)	(0.038, 0.227)	(0.050, 0.313)
Difference	Estimate	0.357^*^	0.183^*^	0.183^*^	0.102^*^	0.102^*^
	95%CI	(0.068, 0.609)	(0.033, 333)	(0.033, 0.333)	(0.032, 0.221)	(0.032, 0.221)

*N* = 400, ^*^P < 0.05, ^**^P < 0.01, ^***^P < 0.001, Bootstrap = 5,000.

In addition, the analysis results of Model 7 in PROCESS v4.3 for this moderated mediation model showed: (1) The direct effect of AI empathy perception on employees' prosocial behavior was 0.080, with a 95% confidence interval of (−0.019, 0.180), including 0, indicating that it was fully mediated; (2) The mediating effect of warmth between AI empathy perception and employees' prosocial behavior was 0.158, with a 95% confidence interval of (0.063, 0.263), excluding 0, suggesting that the mediating effect of warmth was significant; (3) The interaction effect between AI empathy perception and AI anthropomorphism on warmth was 0.208, with a 95% confidence interval of (0.138, 0.279), excluding 0, indicating that AI anthropomorphism positively moderated the relationship between AI empathy perception and warmth; (4) The moderating effect of AI anthropomorphism in the relationship between AI empathy perception and employees' prosocial behavior through warmth was 0.060, with a 95% confidence interval of (0.008, 0.110), excluding 0, suggesting that an increase in AI anthropomorphism enhanced the effect of AI empathy perception on employees' prosocial behavior through warmth. In summary, Hypotheses 1, 2, 3, and 4 were all supported.

## 5 Discussion

AI was endowed with empathy, enabling it to understand, perceive, and respond to human emotions and needs. Previous research has confirmed that AI empathy is associated with people's attitudes toward it (Xue et al., 2023). However, due to stereotypes, people often perceive AI as lacking emotional warmth and tend to ignore its social presence. This seems to pose a significant challenge for managers and developers. Our study aimed to provide a reasonable explanation for “the association between AI empathy perception, warmth, and employees' prosocial behavior in the workplace and to help people understand the behavioral shifts that AI, as a partner, brings to human individuals emotionally and cognitively. Based on social cognitive theory, we established a theoretical model and proposed four research hypotheses. Under the model associated with enhanced human employees' prosocial behavior, we explored the intrinsic associations of warmth and AI anthropomorphism, following a research approach of “background analysis—literature review to propose research questions—constructing a theoretical model to derive hypotheses—empirical research—inductive and deductive verification of the theoretical model—analysis of conclusions and applications—future prospects.” Empirical analysis revealed that when designing AI images and applications, in addition to efficiency-driven capabilities, endowing AI colleagues with appropriate anthropomorphic styles and social-emotional expressions could be linked to increased human employees' prosocial behavior. This study combined AI's empathy capabilities with anthropomorphism technology to explore the association between them and employees' prosocial behavior and validated the mediating role of warmth in this process within the framework of social cognitive theory. The evolution of AI has been observed over time, with discussions on human-computer interaction becoming progressively more intense in the literature. The integration of AI into the workplace is a gradual process, and the fusion of humans and AI has only recently begun. Research on this topic in academia is still somewhat fragmented. To gain a comprehensive understanding of the relationship between human employees and AI's empathy and anthropomorphic traits, researchers from diverse fields must continue to make strides in both theoretical and practical areas. The following outlines the theoretical implications, practical insights, and the limitations and future directions of our study.

### 5.1 Theoretical implications

The theoretical implications of this study are as follows:

First, by contextualizing the warmth–competence framework (Fiske et al., 2007) in AI–employee collaborative settings, this study extends the general understanding of AI empathy by empirically verifying its unique mechanism in shaping workplace prosocial behavior. Unlike prior research that focuses on AI empathy in broad contexts (e.g., customer service), we specifically demonstrate that employees' perceived AI empathy—manifested through warmth expressions such as emotional validation and personalized support—facilitates their prosocial behavior by enhancing psychological emotion regulation. Furthermore, this study identifies perceived AI warmth as a critical mediator linking AI empathy to employee acceptance. When employees perceive AI as warm, their trust in and acceptance of AI are significantly strengthened, which in turn motivates prosocial behavior (Harris-Watson et al., 2023). This finding enriches the theoretical model of AI acceptance by incorporating an emotional-social dimension, moving beyond traditional technology acceptance frameworks that emphasize functional attributes (e.g., perceived usefulness). It also provides theoretical guidance for organizational managers to optimize AI deployment strategies by prioritizing warmth-oriented design.

Second, this study addresses the theoretical gap in understanding whether AI—often stereotyped as cold and objective (Longoni et al., 2019)—can genuinely elicit employees' perceptions of care and understanding, thereby extending Inzlicht et al.'s (2024) research on the recipient experience of AI empathy. We clarify that AI warmth, as a core component of the social-cognitive dimension of AI empathy, enables AI to simulate human emotional perception and transmission. Through contextually adaptive empathetic expressions, AI conveys care and support to employees, triggering positive emotions and reinforcing the perception of being understood. This process not only explains why AI empathy promotes prosocial behavior but also reveals AI's potential to enhance human well-being in the workplace—a novel theoretical insight that expands the boundaries of AI emotional intelligence research. By bridging the literature on human-AI emotional interaction and the emotion-behavior link in behavioral science, this study provides a new theoretical perspective for understanding employees' behavioral motivations in the AI-augmented workplace, with implications for organizational behavior and management research.

Finally, this study contributes to the literature by uncovering the moderating role of AI anthropomorphism in the relationship between perceived AI empathy (via warmth) and employees' prosocial behavior. We find that moderate AI anthropomorphism reverses the “cold technology” bias by fostering a sense of social presence (Janson, 2023), which enhances the authenticity of employees' perceived AI warmth. This, in turn, amplifies the positive impact of AI empathy on prosocial behavior. Unlike prior studies that focus on the direct effects of AI anthropomorphism on user attitudes, we delve into its cognitive mechanism—specifically, how it shapes the processing of AI's warm expressions and strengthens the empathy-prosocial behavior link. This finding refines the theoretical understanding of the boundary conditions of AI empathy's effectiveness and provides a nuanced explanation for when and how AI can effectively influence human social behavior in organizational contexts.

### 5.2 Practical implications

Firstly, during employee training and organizational culture shaping, organizations and managers need to properly guide human employees' attitudes toward AI partners, instilling concepts conducive to human-AI collaboration and enhancing human employees' ability to perceive AI empathy. Previous studies have shown that collaborating with AI can be associated with increased empathetic abilities among human collaborators (Sharma et al., 2023), and the cognitive scalability and complexity of AI systems can continuously provide the necessary empathy without considering future emotional returns, while avoiding the empathy “burnout” that humans might experience. Since AI can maintain and operate emotional simulations for a certain number of agents simultaneously, it may have the potential to facilitate empathetic behaviors at a scale beyond the capabilities of individual or collective humans (Christov-Moore et al., 2023). In conjunction with our findings, human employees' perception of empathetic behavior arising from the interaction between their cognition and emotions with AI contributes to the creation of a positive work atmosphere, strengthens emotional connections between team members, and further facilitates more prosocial behavior from employees. Therefore, if managers properly guide employees' attitudes toward AI, this outcome will likely help promote team cooperation and collaboration, supporting improvements in team performance and creativity.

Secondly, establishing a human-AI collaboration capability development system, and using cognitive restructuring training to help employees develop “digital emotional intelligence,” can position AI empathy as a catalyst for fostering human employees' prosocial behavior and organizational cohesion. Human employees should develop the ability to utilize AI-driven empathy expressions to ease negative emotions and enhance emotional intelligence, thereby boosting their own competitiveness. The way employees perceive AI empathy—particularly the warm cognitive feelings tied to an enriched emotional atmosphere—can help reduce the threats, stress, and discomfort they may feel toward AI. In today's world, many individuals worry that AI might replace human workers, leading to negative and resistant attitudes toward the integration of AI in the workplace. Consequently, during human-AI interactions, AI empathy can play a crucial role in fostering positive emotions related to care, belonging, and recognition, helping to counterbalance some of these negative effects. Meanwhile, managers should guide employees to understand the importance of warmth and emotional intelligence through AI empathy demonstrations, helping them develop “digital emotional intelligence” and, in turn, encouraging their prosocial behavior, such as stronger empathy, self-growth, and enhanced personal competitiveness, thus supporting their ability to meet the ever-changing demands of the real-world environment (Gorny et al., 2023).

Finally, further development of emotional interaction modules is necessary to enhance the realism and emotional resonance of human-AI interaction. In AI design, it is important to appropriately use anthropomorphic features and more accurate emotional recognition algorithms, as well as personalized and humanized emotional feedback mechanisms, while avoiding the uncanny valley effect. This can be achieved through natural language processing and facial emotion recognition to enable empathetic conversations, thus increasing human employees' trust and acceptance of AI. Considering the remarkable capabilities of AI and the growing depth of human-AI interactions, the relationship has progressively evolved from human-AI integration to human-AI symbiosis (Zhang and Wang, 2024). As a result, the design and implementation of AI anthropomorphism in work interfaces are expected to become increasingly refined. Additionally, anthropomorphized AI may facilitate more social connections, making human employees emotionally and cognitively trust anthropomorphized intelligence, and possibly even generating a sense of effectiveness and capability in interactions with AI (Johnson, 2024). Therefore, developers can enhance AI's empathy by creating more precise emotional recognition algorithms and designing more personalized emotional feedback mechanisms that allow AI to better understand human employees' emotional states (Shen et al., 2025). Coupled with the moderating effect of anthropomorphic design, AI can more accurately simulate human emotional interactions, promoting human employees' acceptance and emotional connection with AI. This emotional connection contributes to employees feeling a stronger sense of capability and effectiveness in their work. However, since AI is not a human entity, it can respond to employees' positive emotions but cannot provide a sense of value when employees engage in altruistic and reciprocal prosocial behavior. Furthermore, human employees' expertise or work skills in AI-related fields are crucial to their psychological outcomes (Jia et al., 2024). Therefore, managers should offer targeted training to guide employees' positive emotional orientation and enhance their willingness and ability to engage in prosocial behavior by improving AI's understanding and response to employees' emotional needs.

## 6 Limitations and future research directions

This study has four key limitations that require explicit elaboration: First, all variables—including the core dependent variable of prosocial behavior—are measured via self-report questionnaires. Although procedural controls (e.g., anonymous completion, item randomization, reverse scoring) have been implemented to mitigate biases, subjective reports inherently cannot fully eliminate social desirability and cognitive biases. Furthermore, they only capture prosocial intentions and self-perceived behaviors rather than actual behavioral performance in real work settings, resulting in limited ecological validity and failure to verify the “intention-behavior” transformation chain. Second, the measurement of AI warmth expressions relies on employees' subjective ratings, lacking objective quantitative indicators derived from AI interaction outputs. This not only fails to accurately reflect the true characteristics and intensity of AI warmth expressions but also overlooks the academic suggestion of “sing LLMs to measure AI warmth expressions,” thereby compromising the accuracy of core construct measurement. Third, the research sample is confined to digital firms in China. Such samples are characterized by high technology acceptance and advanced organizational digital maturity, while being shaped by China's collectivist cultural context. These factors may impede the generalization of findings to other cultural contexts (e.g., Western individualistic cultures) or non-digital organizational settings (e.g., traditional manufacturing enterprises, public service institutions), restricting the external validity of the results. Fourth, the study does not systematically explore the specific mechanisms through which single-source questionnaire measurement undermines ecological validity, nor does it propose feasible schemes for integrating behavioral or computational measures, indicating a lack of in-depth reflection on methodological limitations.

To address the aforementioned limitations, future research can make systematic improvements in four aspects: First, adopt a multi-method and multi-source data collection strategy to enhance ecological validity. Construct simulated work scenarios (e.g., cross-departmental collaboration, emergency task support) through behavioral experiments to directly observe and document employees' actual prosocial behaviors (e.g., duration of active assistance, quality of collaborative contributions). Incorporate third-party indicators such as peer evaluations and objective organizational data (e.g., collaboration completion rates, records of voluntary participation in additional work) to establish a dual verification system of “subjective intentions-objective behaviors.” Second, respond to reviewers' suggestions by developing an objective quantitative tool for AI warmth assessment. Leverage natural language processing (NLP) technology and AI's text analysis capabilities to build an automated evaluation framework: train a dedicated LLM scoring model using a labeled corpus encompassing dimensions such as emotional empathy and personalized responses, then conduct standardized scoring of target AI interaction outputs. This achieves the precise “measurement of AI warmth expressions by LLMs” and reduces subjective biases. Third, expand samples and contexts to improve external validity. Adopt a cross-cultural and cross-industry multi-case research design, incorporating samples from diverse cultural backgrounds (e.g., Chinese vs. Western) and varying levels of digital maturity (e.g., traditional industries vs. emerging technology enterprises). Conduct comparative analyses of the moderating effects of cultural values (e.g., collectivism vs. individualism) and organizational digitalization levels on the “AI warmth expressions-employees' prosocial behavior” relationship to test the generalizability of research conclusions. Fourth, deepen methodological integration and mechanism exploration. Adopt a mixed-methods research approach, integrating objective AI data, employees' subjective perceptions, and actual behavioral data to dissect the chain mechanism of “objective warmth characteristics-subjective perception-behavioral transformation.” Additionally, design longitudinal studies to observe the dynamic evolutionary relationships between variables over time, incorporating contextual variables such as task complexity and organizational climate to provide more robust empirical support for the theoretical model.

## 7 Conclusion

One of the most important design factors to consider for successful human-AI interaction is how the characteristics of AI members influence human employees' acceptance of AI. Our research, following the finding that AI empathy perception and warmth increase human acceptance, further confirms the impact of AI empathy and warmth on human employees' prosocial behavior, with appropriate anthropomorphism playing a positive moderating role in this process. The results emphasize the importance of creating empathetic AI and the need for a balanced level of anthropomorphism. They also highlight the necessity of exploring the effects of AI empathy, or its perception, on other work processes. Future studies should prioritize both human-centered and technology- or tool-centered development approaches, as the integration of these two perspectives, both in theory and practice, can significantly enhance organizational performance.

## Data Availability

The raw data supporting the conclusions of this article will be made available by the authors, without undue reservation.
